# The optimal cut‐off value in fit‐based colorectal cancer screening: An observational study

**DOI:** 10.1002/cam4.3761

**Published:** 2021-02-03

**Authors:** Sisse Helle Njor, Berit Andersen, Lennart Friis‐Hansen, Niels de Haas, Dorte Linnemann, Henrik Nørgaard, Ole Roikjær, Bo Søndergaard, Morten Rasmussen

**Affiliations:** ^1^ Department of Public Health Programmes Randers Regional Hospital Randers Denmark; ^2^ Department of Clinical Medicine Aarhus University Aarhus Denmark; ^3^ Danish Colorectal Cancer Screening Database (DCCSD) Steering Committee Aarhus Denmark; ^4^ Department of Clinical Biochemistry Hilleroed Hospital Hillerød Denmark; ^5^ Department of Gastroenterology Aalborg University Hospital Aalborg Denmark; ^6^ Department of Pathology Herlev and Gentofte Hospital Herlev Denmark; ^7^ Department of Radiology Herlev Hospital Herlev Denmark; ^8^ Department of Surgery Zealand University Hospital Roskilde Denmark; ^9^ Gastrounit Medical Division Hvidovre University Hospital Hvidovre Denmark; ^10^ Bispebjerg University Hospital Copenhagen Denmark

**Keywords:** capacity building, colorectal neoplasms, mass screening, sensitivity, specificity

## Abstract

**Background:**

Colorectal cancer (CRC) screening programs using fecal immunochemical test (FIT) have to choose a cut‐off value to decide which citizens to recall for colonoscopy. The evidence on the optimal cut‐off value is sparse and based on studies with a low number of cancer cases.

**Methods:**

This observational study used data from the Danish Colorectal Cancer Screening Database. Sensitivity and specificity were estimated for various cut‐off values based on a large number of cancers. Traditionally optimal cut‐off values are found by weighting sensitivity and specificity equally. As this might result in too many unnecessary colonoscopies we also provide optimal cut‐off values for different weighting of sensitivity and specificity/number of needed colonoscopies to detect one cancer.

**Results:**

Weighting sensitivity and specificity equally gives an optimal cut‐off value of 45 ng Hb/ml. This, however, means making 24 colonoscopies to detect one cancer. Weighting sensitivity lower and for example, aiming at making about 16 colonoscopies to detect one cancer, gives an optimal cut‐off value of 125 ng Hb/ml.

**Conclusions:**

The optimal cut‐off value in an FIT population‐based screening program is 45 ng Hb/ml, when as traditionally sensitivity and specificity are weighted equally. If, however, 24 colonoscopies needed to detect one cancer is too huge a burden on the health care system and the participants, 80, 125, 175, and 350 ng Hb/ml are optimal cut‐off values when only 19/16/14/10 colonoscopies are accepted to find one cancer.

## Graphical abstract

If 24 colonoscopies needed to detect one cancer is acceptable for the health care system and the participants, the optimal cut‐off value in fecal immunochemical test screening is 45 ng Hb/ml. When only 19/16/14/10 colonoscopies are accepted to find one cancer, then, the optimal cut‐off value is 80/125/175/350 ng Hb/ml.

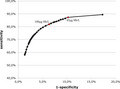

**Lay Summary**

Studies with relatively few cancers have estimated the optimal cut‐off value in FIT‐based CRC screening to be 50–150 ng Hb/ml when, as traditionally, weighting sensitivity, and specificity equally.

It can be discussed whether it is acceptable to weight sensitivity and specificity equally as this results in many unnecessary colonoscopies to detect one cancer.

No studies have estimated optimal cut‐off value without weighting sensitivity and specificity equally.

This study used different weighting of sensitivity and specificity and found that given it is acceptable to make 24/19/16/14/10 colonoscopies to detect one cancer, the optimal cut‐off value is 45, 80, 125, 175, and 350 ng Hb/ml.

## 1 INTRODUCTION

In order to detect colorectal cancer (CRC) at a precancerous or early stage, where it is curable, several countries and regions have started population‐based CRC screening. Different methods/technologies are used to screen people for CRC. Since colorectal adenomas and tumors often bleed,^1^ one possible screening method is the fecal immunochemical test (FIT), which detects tiny amounts of human hemoglobin in a stool sample.

Screening should sort out apparently healthy persons who probably have a disease from those who probably do not.^2^ In FIT screening the sorting is done by allocating participants with an FIT level above a certain cut‐off value (positive screening test) to the group who probably have CRC, leaving those with an FIT level below a certain cut‐off value (negative screening test) in the group who probably do not have CRC.

The cut‐off value should be chosen so that the odds of having a positive screening test given that you have the disease (sensitivity) is as high as possible, while at the same time maximizing the odds of having a negative screening test given that you do not have the disease (specificity). Decreasing the cut‐off value to get a higher sensitivity will though inevitably decrease specificity. The optimal cut‐off value, therefore, depends upon how important an increase in sensitivity is weighted compared to the inevitable following decrease in specificity.

Six studies have previously reported sensitivity and specificity for various cut‐off values.^3^, ^4^, ^5^, ^6^, ^7^, ^8^ However, all of these studies are based on a low number of CRCs and none of the studies are from population‐based screening programs.

This register‐based study aims at estimating sensitivity and specificity for various cut‐off values in the prevalence round of a population‐based FIT screening program with a large number of CRCs, and thereby provide optimal cut‐off values for various weighting of sensitivity and specificity.

## 2 METHODS

### 2.1 The Danish CRC screening program

Population‐based CRC screening with FIT started in Denmark in March 2014 for citizens aged 50–74 years. In order to gradually build up the needed capacity in hospitals, the first screening round took almost 4 years and ended in December 2017. All citizens aged 50–74 years at some point in 2014–2017 were invited to participate in the first screening round.^9^ Citizens already enrolled in a surveillance program after a CRC or adenoma diagnosis, were advised in the invitation letter not to participate. Citizens with inflammatory bowel disease were advised to discuss with their treating physician whether participation was relevant. Returned FITs were analyzed using the OC Sensor Diana (2013–2017) (Eiken Chemical Company), yielding how much hemoglobin the sample contains. A test was considered positive if the stool‐sample contained more than 100 ng hemoglobin (Hb)/ml (20 µg Hb/g feces as the sample bottle collects 10 mg feces and contains 2 ml buffer). Citizens with a positive FIT were informed by letter and referred to colonoscopy at a hospital‐based screening endoscopy unit.

### 2.2 Study design and population

Our study population consists of all participants in the Danish CRC screening program who submitted an analyzable stool‐sample between 1 March 2014 and 31 December 2015. The study population was followed for CRC diagnoses for 2 years after the sample was analyzed.

### 2.3 Data

Information on invitation date, date when returned sample was analyzed and the amount of blood in the sample was retrieved from the Danish CRC Screening Database (DCCSD),^10^ a clinical quality database that was established to monitor the quality of the national Danish CRC screening program. FIT‐values below 35 ng Hb/ml were only known as ≤35 ng Hb/ml and FIT‐values above 1000 ng Hb/ml were only known as ≥1000 ng Hb/ml. DCCSD gets individual data on all invitations and participation from the administrative database IAM.^11^ Information on CRCs, adenomas, and colonoscopies was obtained from the Danish National Pathology Data Bank and the Danish National Patient Register. Data were linked using the unique Danish personal registration number issued to all Danish residents. The completeness and reliability of the Danish National Pathology Data Bank and the Danish National Patient Register is deemed to be very high and good.^12^, ^13^

### 2.4 Definitions

We identified CRCs as samples from the colon or rectum with a SNOMED code M8*3 (cancer). Adenoma(s) were identified as samples from the colon or rectum with one of these SNOMED codes: M8213F (flat adenoma), M82110 (tubular adenoma), M82130 (traditional serrated adenoma), M8213M (sessile serrated lesion with dysplasia), M82630 (tubulovillous adenoma), and M82611 (villous adenoma).

We defined a participant as a citizen who sent in an analyzable stool‐sample in the study period (1 March 2014 and 31 December 2015). A positive FIT is a FIT where the measured amount of blood is above the cut off level. We estimated the number of true positive/false positive FITs by the number of positive FITs where the participant is diagnosed/not diagnosed with CRC 0–24 months after the test. Similarly the number of false negative/true negative FITs was estimated by the number of nonpositive FITs where the participant is diagnosed/not diagnosed with CRC 0–24 months after the test. We defined Sensitivity for the FIT test as the number of true positives divided by the number of true positives plus the number of false negatives. Specificity was defined as the number of true negatives divided by the number of true negatives plus the number of false positives.

### 2.5 Analyses

In order to avoid that the number of cancers within each group of FIT values were too low, the span of the analyzed cut‐off values was 5 ng Hb/ml for cut‐off values between 35 and 75 ng Hb/ml; 10 ng Hb/ml for cut‐off values between 80 and 100 ng Hb/ml; 25 ng Hb/ml for cut‐off values between 100 and 500 ng Hb/ml and 100 ng Hb/ml for cut‐off values between 500 and 1000 ng Hb/ml.

**FIGURE 1:**
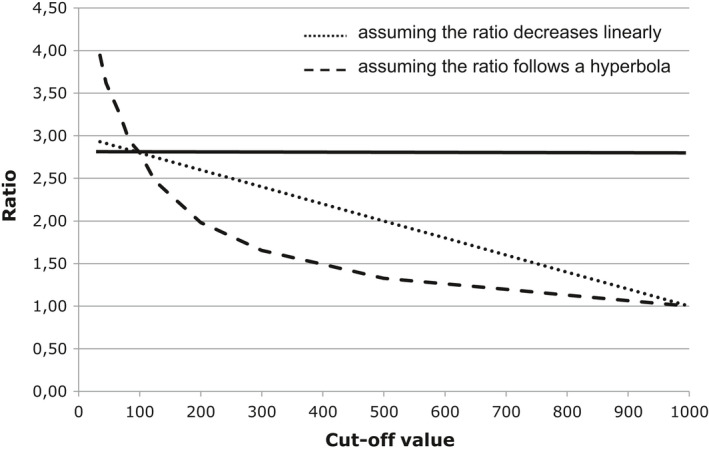
The assumed ratio between number of true positive cases and number of false negative cases for cut‐off values 0–1000 ng Hb/ml

In the optimal study design all participants would have had a subsequent colonoscopy. This was, however, not the case in this observational study. As many of the cancers are at an early stage when discovered by screening it is not likely that all true positive cases with a cut‐off value between 100 ng Hb/ml and 100 + *x* ng Hb/ml would have emerged as cancers in 2 years after the test, had the cut‐off value been 100 + *x* ng Hb/ml. As we estimate number of false negative cases by number of negative test, which emerges as cancer in 2 years after the test, we did not know how many false negative cases there would have been, had the cut‐off value been above 100 ng Hb/ml. We neither know how many true positive cases there would have been, had the cut‐off value been below 100 ng Hb/ml, as these participants had no colonoscopy. There are 18.04 true positive cases per 1000 tests among tests with a cut‐off value of 100–149 ng Hb/ml, whereas there are 6.35 false negative cases per 1000 tests among tests with a cut‐off value of 80–99 ng Hb/ml. Close to 100 ng Hb/ml the ratio between number of true positive cases and number of false negative cases is, therefore, approximately 2.8 (18.04/6.35). In our main analysis, we assumed that the ratio between number of true positive cases and number of false negative cases was 2.8 for all cut‐off values. The ratio between the number of true positive cases and the number of false negative cases might though be lower for high FIT values than for low FIT values. We, therefore, made two sensitivity analyses, one assuming the ratio to decrease linearly (dotted line, Figure 1) and one assuming the ratio to follow a hyperbola (dashed line, Figure 1).

A receiver operating characteristic curve (ROC curve) was used to show the trade‐off between sensitivity and specificity.^14^ The Youden index, that is, sensitivity + specificity, is a commonly used measure of overall diagnostic effectiveness.^15^ Traditionally the optimal cut‐off point is found by putting equal weight on sensitivity and specificity and thereby maximizing sensitivity + specificity (the Youden index). We not only calculated the optimal cut‐off value using this method, but also recognized that it might not be appropriate in CRC screening to weight sensitivity and specificity equally. The current cut‐off value (100 ng Hb/ml) resulted in 495,785 true negative, 33,496 false positive, 2086 true positive and 461 false negative, giving a specificity of 93.7% and 81.9% sensitivity. Decreasing specificity by 1–92.7% would result in almost 5000 cancer‐free citizens referred unnecessarily to colonoscopy (1% of about 500,000 participants without cancer). Increasing sensitivity by 1–82.9% would result in 25 extra citizens having their cancer detected at screening (1% of about 2500 participants with cancer). Whether 5000 unnecessary colonoscopies (1% change in specificity) should be weighted equally to 25 extra cancers detected (1% change in sensitivity) is a decision to make in each screening program. If the burden of unnecessary colonoscopies on the health care system and the participants should be weighted higher then specificity should be given more weight than sensitivity. We, therefore, also calculated the Youden index giving different weighting to specificity versus sensitivity. To visualize how these different weighting of sensitivity versus specificity affects number of screen‐detected CRC and number of needed colonoscopies, we for each optimal cut‐off value also calculated percentage CRCs missed/detected and percentage less/extra colonoscopies needed compared to a cut‐off of 100 ng Hb/ml.

The 10‐year average risk that an advanced adenoma transition to CRC has been reported to be 25–40%.^16^ Although it is important to detect adenomas at CRC screening it is not equally important as finding CRCs. It is, therefore, not appropriate to include the number of screen‐detected adenomas when calculating sensitivity and specificity. To show how the number of detected adenomas is affected by choice of cut‐off value, we calculated percentage missed adenomas compared to a cut‐off value of 100 ng Hb/ml for each optimal cut‐off value.

Data were processed using SAS (version 9.4).

## 3 RESULTS

**FIGURE 2:**
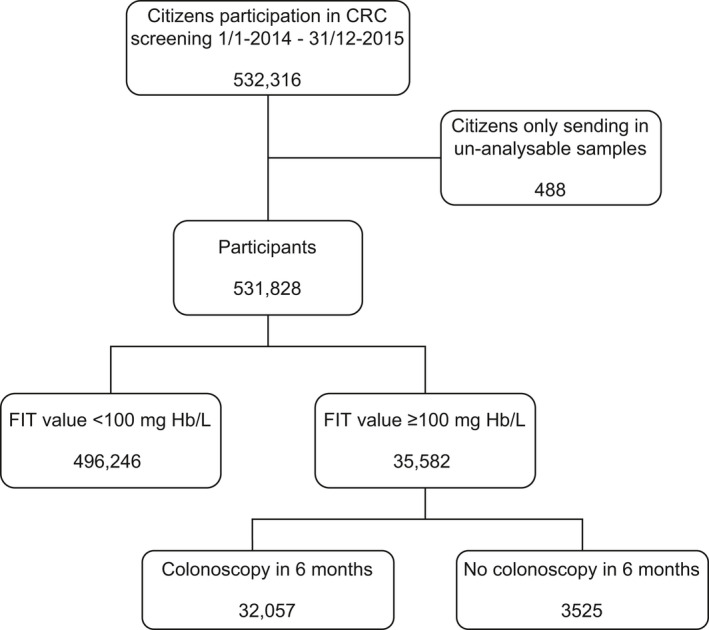
Flowchart of study population and exclusions

In total 532,316 citizens accepted the invitation to CRC screening and sent in a stool sample before 31 December 2015. Of these 488 only sent in un‐analyzable stool samples and were, therefore, excluded from the analyses (Figure 2). Among the remaining 531,828 eligible participants 35,582 had a FIT value above 100 ng Hb/ml and were, therefore, invited to colonoscopy. Of these 90.1% (32,057) had a colonoscopy within 6 months (Figure 2).

At the current cut‐off value of 100 ng Hb/ml, screening of 531,828 citizens resulted in 2086 screen detected cancers (true positive cases), 33,496 false positive cases, 495,785 true negative cases and 461 interval cancers (false negative cases). Table 1 shows that Increasing the cut‐off value from 100 to 200 ng Hb/ml will decrease the sensitivity from 81.9% [95% CI: 80.8–83.0%] to 77.0% [95% CI: 75.7–78.2%] and increase the specificity from 93.7% [95% CI: 93.6–93.7%] to 95.9% [95% CI: 95.8–95.9%].

**TABLE 1:** Sensitivity and specificity for different cut‐off values

Cut‐off value	Sensitivity	Specificity
40	89.5% [88.7–90.3%]	82.7% [82.6–82.8%]
45	87.3% [86.4–88.2%]	89.8% [89.7–89.8%]
50	86.5% [85.6–87.4%]	90.4% [90.3–90.4%]
55	85.9% [84.9–86.9%]	90.9% [90.8–90.9%]
60	85.5% [84.5–86.5%]	91.3% [91.2–91.3%]
65	84.8% [83.8–85.8%]	91.7% [91.7–91.8%]
70	84.0% [83.0–85.1%]	92.1% [92.1–92.2%]
75	83.8% [82.7–84.8%]	92.5% [92.5–92.6%]
80	83.4% [82.4–84.5%]	92.8% [92.7–92.8%]
90	82.6% [81.5–83.7%]	93.3% [93.2–93.3%]
100	81.9% [80.8–83.0%]	93.7% [93.6–93.7%]
125	80.7% [79.5–81.8%]	94.4% [94.3–94.4%]
150	79.5% [78.3–80.7%]	94.9% [94.9–95.0%]
175	78.4% [77.2–79.6%]	95.4% [95.4–95.5%]
200	77.0% [75.7–78.2%]	95.9% [95.8–95.9%]
225	75.5% [74.2–76.8%]	96.2% [96.2–96.3%]
250	74.4% [73.0–75.7%]	96.5% [96.5–96.5%]
275	73.5% [72.1–74.8%]	96.7% [96.7–96.8%]
300	72.7% [71.3–74.0%]	96.9% [96.9–97.0%]
325	71.8% [70.3–73.2%]	97.1% [97.1–97.1%]
350	71.0% [69.5–72.4%]	97.3% [97.2–97.3%]
375	70.1% [68.6–71.5%]	97.4% [97.4–97.4%]
400	69.4% [67.9–70.9%]	97.5% [97.5–97.6%]
425	68.5% [67.0–70.0%]	97.6% [97.6–97.7%]
450	67.5% [66.0–69.0%]	97.7% [97.7–97.8%]
475	66.8% [65.3–68.4%]	97.8% [97.8–97.8%]
500	65.9% [64.3–67.5%]	97.9% [97.9–97.9%]
600	63.3% [61.7–64.9%]	98.2% [98.1–98.2%]
700	60.8% [59.1–62.4%]	98.4% [98.3–98.4%]
800	58.6% [56.9–60.2%]	98.5% [98.5–98.5%]
900	57.1% [55.4–58.8%]	98.6% [98.6–98.6%]
1000	55.8% [54.1–57.5%]	98.7% [98.7–98.7%]

**FIGURE 3:**
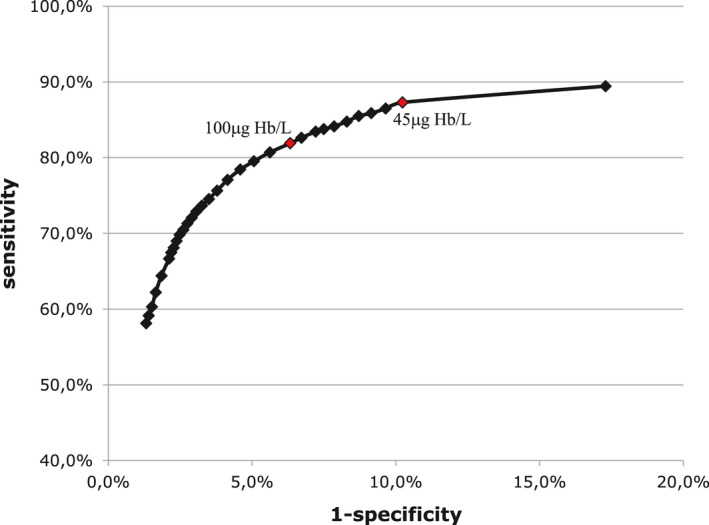
ROC curve for various cut‐off values of FIT

The ROC curve (Figure 3) shows that a cut‐off value of 45 ng Hb/ml result in a point on the ROC curve closest to (0,1), that is, maximizes the Youden index (0.873 + 0.898 = 1.771). The present cut‐off value of 100 ng Hb/ml results in a slightly lower Youden index (0.819 + 0.937 = 1.756)/further distance to (0,1). Lowering the cut‐off value to 45 ng Hb/ml will, however, result in 24 colonoscopies needed to detect one cancer (Table 2). If this is considered to be a too high burden on the health care system and the participants, sensitivity needs to be weighted lower than specificity.

**TABLE 2:** The optimal cut‐off value for different weighting of sensitivity and specificity

Weight sensitivity (%)	Weight specificity (%)	Optimal cut‐off value in ng Hb/ml^a^, ^b^	Optimal cut‐off values in ng Hb/ml^c^	Number needed to scope to find one cancer	% adenomas missed compared to cut‐off 100	% cancers missed/extra compared to cut‐off 100	% less/extra colonoscopies compared to cut‐off 100
50	50	45	45	24	NA	14–17 extra	59 extra
45	55	60	60	21	NA	9–10 extra	36 extra
40	60	80	80	19	NA	4 extra	13 extra
35	65	125	125	16	8	3 missed	11 less
30	70	150	150	15	16	6 missed	19 less
25	75	175	175	14	22	8 missed	26 less
20	80	275	200	11/13^d^	41/28^d^	19/12^d^ missed	47/33^d^ less
15	85	350	300	10/11^d^	49/44^d^	23/20^d^ missed	55/50^d^ less
10	90	475	400	9/10^d^	57/53^d^	30/26^d^ missed	63/59^d^ less

^a^ Assuming a constant ratio between number of false negative cases and number of true positive cases of 2.8 (solid line, Figure 1).

^b^ Assuming the ratio between number of false negative cases and number of true positive cases decreases linearly (dotted line, Figure 1).

^c^ Assuming the ratio between number of false negative cases and number of true positive cases follow a hyperbola (dashed line, Figure 1).

^d^ When using the optimal cut‐off values from the “hyperbola” sensitivity analyses.

Table 2 shows the optimal cut‐off value for different weighing of sensitivity and specificity. Weighting sensitivity by 40% and specificity by 60% (i.e., maximizing 0.4 × sensitivity+0.6 × specificity) and thereby accepting to make 19 colonoscopies to detect one cancer gives an optimal cut‐off value of 80 ng Hb/ml. Using 80 ng Hb/ml as cut‐off value instead of 100 ng Hb/ml will detect 4% extra cancers at the cost of 13% extra colonoscopies. Weighting sensitivity by 35% and specificity by 65% and thereby accepting to make 16 colonoscopies to detect one cancer gives an optimal cut‐off value of 125 ng Hb/ml. The effect of using 125 ng Hb/ml as cut‐off value instead of 100 ng Hb/ml is 11% less colonoscopies needed as well as 8% less adenomas and 3% less cancers detected. As seen in Table 2, the two sensitivity analyses resulted in unchanged optimal cut‐off values except when sensitivity is weighted by only 10–20%.

## 4 DISCUSSION

The study provided optimal cut‐off values for FIT‐based CRC screening for various weighing of sensitivity versus specificity. Sensitivity analyses revealed that the provided optimal cut‐off values were quite robust toward changes to the assumptions.

### 4.1 Strengths and weaknesses

The major strengths of this study are the large number of participants and subsequent cancers as well as the use of high validity Danish registers to follow all participants on an individual level for 2 years.

We only included cancers diagnosed up to 24 months after the FIT to decide whether the test was true positive, false positive, false negative or true negative. This can have caused some misclassification, for example, citizens with a positive FIT and a CRC not found at colonoscopy nor within the next 2 years, will be falsely categorized as false positive although they are true positive. This number is though low, as number of interval cancers after a clean colon colonoscopy in the Danish CRC screening program is low.^17^ As seen in our sensitivity analyses small changes to number of true/false positive and true/false negative cases only result in minor changes to the optimal cut‐off values, wherefore the effect of these misclassifications is expected to be minimal.

As only participants with a cut‐off value above 100 ng Hb/ml had a subsequent colonoscopy, we had to estimate number of false negative cases had the cut‐off value been above 100 ng Hb/ml and number of true positive cases had the cut‐off value been below 100 ng Hb/ml. Our sensitivity analyses though showed that the results are quite robust toward major changes to our assumptions on the ratio between number of true positive cases and number of false negative cases.

### 4.2 Other studies

Previously six studies have reported on sensitivity and specificity for various cut‐off values.^3^, ^4^, ^5^, ^6^, ^7^, ^8^ The numbers of cancers were quite low in all five studies, ranging from 8 to 89 cancers. In comparison our study included 2251 cancers.

Weighting sensitivity and specificity equally, Itoh et al., Launoy et al., and de Wijkerslooth et al. found an optimal cut off value of 50 ng Hb/ml.^3^, ^4^, ^5^ Brenner et al. like us found the optimal cut off value to be 45 ng Hb/ml.^8^ The study by Wijkerslooth used the same test as we did (OC‐Sensor; Eiken Chemical Company), while the other studies used the OC‐hermodia test (Eiken Chemical Company), the Magstream 1000 test (Fujirebio Inc.), or the FOB‐Gold test (Sentinel Diagnostics). Using the OC‐Sensor test (Eiken Chemical Company) and weighting sensitivity and specificity equally Chen et al. found the optimal cut off value, to be 100 ng Hb/ml and Aniwan et al. found the optimal cut off value to be 150 ng Hb/ml.^6^, ^7^ Chen et al. favored a cut off value of 100 ng Hb/ml above 50 ng Hb/ml as their study had no cancers among FITs with an FIT value between 50 and 100 ng Hb/ml, resulting in unchanged sensitivity for cut off values between 50 and 100 ng Hb/ml.^6^ Similarly Aniwan et al. favored 150 ng Hb/ml over 50 ng Hb/ml as their study had no cancers among FITs with an FIT value between 50 and 150 ng Hb/ml, resulting in unchanged sensitivity for cut off values between 50 and 150 ng Hb/ml.^7^

Our results, when weighting sensitivity and specificity equally, are in line with results from all of these studies, suggesting that they are generalizable to other types of FIT. Brenner et al. calculated sensitivity and specificity both for detecting CRC and advanced neoplasms and found the same optimal cut‐off value.^8^

To show results based on real‐life data from an organized CRC screening program, we also included citizens who chose not to have the recommended colonoscopy after a FIT value ≥100 ng Hb/ml. In the Danish CRC screening program about 90% had a colonoscopy after a positive FIT. Programs with a lower colonoscopy participation rate might experience “Number needed to scope to find one cancer” different from those reported here.

Different cut‐off values males and females might be an appropriate consideration for future studies as, for example, the Danish CRC screening program has shown a higher positivity rate and increased detection of CRC among males as compared to females.^17^, ^18^

## 5 CONCLUSION

The optimal cut‐off value in an FIT population‐based screening program is 45 ng Hb/ml when, as traditionally, sensitivity, and specificity are weighted equally. If, however, 24 colonoscopies needed to detect one cancer is too huge a burden on the health care system and the participants, 80, 125, 175, and 350 ng Hb/ml are optimal cut‐off values when only 19/16/14/10 colonoscopies are accepted to find one cancer.

## 6 ETHICS STATEMENT

According to the Consolidation Act on Research Ethics Review of Health Research Projects, Consolidation Act number 1083 of 15 September 2017 section 14 (2) notification of questionnaire surveys or medical database research projects to the research ethics committee system is only required if the project involves human biological material. Therefore, this study may be conducted without an approval from the committees. According to EU's General Data Protection Regulation (article 30), the project was listed at the record of processing activities for research projects in Central Denmark Region (J. no: 1‐16‐02‐68‐20).

## CONFLICT OF INTEREST

Sisse Njor has received a speaking fee for giving a speech in a colorectal cancer symposium sponsored by Norgine. The money obtained through this has not been used to finance this manuscript. Berit Andersen is head of the colorectal cancer screening programme in Central Denmark Region, but has no other conflicts of interest. Lennart Friis‐Hansen was head of the colorectal cancer screening programme in Region Zealand (March 2014–September 2016), but has no other conflicts of interest. Niels de Haas, Dorte Linnemann, Henrik Nørgaard, Ole Roikjær, Bo Søndergaard have no conflict of interest. Morten Rasmussen is head of the colorectal cancer screening programme in the capital Region and has received speaking fees from Norgine and Ferring Pharmaceuticals for giving speeches at sponsored meetings and symposiums. The money obtained through this has not been used to finance this manuscript.

## AUTHOR CONTRIBUTIONS

SHN, LF, and MR made the study concept and design; SHN, BA, LF, NH, DL, HN, OR, BS, and MR participated in the acquisition of data and analysis and interpretation of data. SHN drafted the manuscript and did the statistical analysis. SHN, BA, LF, NH, DL, HN, OR, BS, and MR made critical revision of the manuscript for important intellectual content.

## Data Availability

In accordance with Danish law, data can not be made publicly available. They may be available on request to the corresponding author.
